# Immune Cell Infiltration into the Brain After Ischemic Stroke in Humans Compared to Mice and Rats: a Systematic Review and Meta-Analysis

**DOI:** 10.1007/s12975-021-00887-4

**Published:** 2021-01-26

**Authors:** Carolin Beuker, Jan-Kolja Strecker, Rajesh Rawal, Antje Schmidt-Pogoda, Tobias Ruck, Heinz Wiendl, Luisa Klotz, Wolf-Rüdiger Schäbitz, Clemens J. Sommer, Heike Minnerup, Sven G. Meuth, Jens Minnerup

**Affiliations:** 1 Department of Neurology with Institute of Translational Neurology, University of Münster, Albert-Schweitzer-Campus 1, Gebäude A1, 48149 Münster, Germany; 2 Institute of Epidemiology and Social Medicine, University of Münster, Albert-Schweitzer-Campus 1, Münster, Germany; 3 Department of Neurology, Evangelisches Klinikum Bethel, Bielefeld, Germany; 4 Institute of Neuropathology, University Medical Center of the Johannes Gutenberg-University Mainz, Mainz, Germany

**Keywords:** Ischemic stroke, Immune cell infiltration, Inflammation, Meta-analysis

## Abstract

Although several studies have suggested that anti-inflammatory strategies reduce secondary infarct growth in animal stroke models, clinical studies have not yet demonstrated a clear benefit of immune modulation in patients. Potential reasons include systematic differences of post-ischemic neuroinflammation between humans and rodents. We here performed a systematic review and meta-analysis to summarize and compare the spatial and temporal distribution of immune cell infiltration in human and rodent stroke. Data on spatiotemporal distribution of immune cells (T cells, macrophages, and neutrophils) and infarct volume were extracted. Data from all rodent studies were pooled by means of a random-effect meta-analysis. Overall, 20 human and 188 rodent stroke studies were included in our analyses. In both patients and rodents, the infiltration of macrophages and neutrophils preceded the lymphocytic influx. Macrophages and neutrophils were the predominant immune cells within 72 h after infarction. Although highly heterogeneously across studies, the temporal profile of the poststroke immune response was comparable between patients and rodents. In rodent stroke, the extent of the immune cell infiltration depended on the duration and location of vessel occlusion and on the species. The density of infiltrating immune cells correlated with the infarct volume. In summary, we provide the first systematic analysis and comparison of human and rodent post-ischemic neuroinflammation. Our data suggest that the inflammatory response in rodent stroke models is comparable to that in patients with stroke. However, the overall heterogeneity of the post-ischemic immune response might contribute to the translational failure in stroke research.

**Supplementary Information:**

The online version contains supplementary material available at 10.1007/s12975-021-00887-4.

## Introduction

Stroke is a leading cause of death, long-term disability, and cognitive impairment worldwide [1, 2]. Recanalization of the occluded vessel either by pharmacological treatment with tissue plasminogen activator or by thrombectomy is the only therapeutic option to reduce poststroke brain tissue damage and to improve the clinical outcome [3]. Within the past years, the local post-ischemic inflammatory response, i.e., neuroinflammation, was identified as a key pathophysiological element that contributes to secondary brain damage after stroke [4, 5]. This immune response includes the infiltration of circulating leukocytes as well as the activation of local microglia [4–6]. Extensive experimental evidence in animal models demonstrates that different anti-inflammatory strategies reduce secondary infarct growth and therefore represent a promising therapeutic target [7–9]. Recently, clinical trials have been initiated to test immunomodulatory treatments in patients with stroke [10–13]. Results were, however, controversial: while two small trials testing fingolimod have been promising, larger studies testing natalizumab (Anti-VLA4) and enlimomab (Anti-ICAM-1) did not demonstrate a benefit [14]. Recently, pivotal study design differences between experimental studies and clinical trials were shown to contribute to the stepwise efficacy decline of stroke treatments from experimental studies to phase 3 clinical trials [15]. In addition, the question arises whether systematic differences in the poststroke immune response between humans and rodents might contribute to the translational failure. However, systematic comparisons of post-ischemic neuroinflammation between patients and animal models are lacking. In contrast to the local immune response, differences in blood leukocyte composition between humans and rodents are well established. Circulating neutrophils predominate in humans (50–70%), while in rodents, circulating leukocytes mainly consist of lymphocytes (75–90%) [16]. Besides potential differences between humans and animals, poststroke neuroinflammation and the efficacy of anti-inflammatory treatments differ substantially among commonly used animal stroke models suggesting that this heterogeneity contributes to the translational failure [17, 18]. Altogether, single studies analyzing the post-ischemic immune cell infiltration revealed inconclusive results. Hence, we performed a systematic review and meta-analysis to summarize and compare all available published studies on the spatial and temporal distribution of immune cell infiltration in human and rodent stroke.

## Materials and Methods

### Search Strategy

In accordance with PRISMA guidelines, we searched the database PUBMED from its inception date to February 2019. This strategy included the keywords “cerebral ischemia” OR “stroke” OR “cerebral infarct” AND “leukocytes” OR “lymphocytes” OR “granulocytes” OR “neutrophils” OR “monocytes” OR “macrophages.” We included only articles in English and German. The bibliographies of relevant articles were cross-checked for further articles not referenced in the aforementioned database. We excluded editorials, conference abstracts, and review papers. This study is registered with *PROSPERO, number **CRD42019142603**.

### Inclusion/Exclusion Criteria

We included all studies, in which immune cell infiltration (lymphocytes and/or macrophages and/or neutrophils) after human or rodent stroke was quantified by either immunohistochemistry or flow cytometry. We focused on lymphocytes, macrophages, and neutrophils, which are regarded as the main players in the post-ischemic inflammatory response. Included studies in this meta-analysis and review were based on the following criteria: (1) measurement of the amount of cells per given unit; (2) quantification was performed at a certain time point; and (3) permanent or transient middle cerebral artery occlusion (pMCAO or tMCAO), photothrombotic stroke, or other focal stroke models were performed on rodents. The exclusion criteria were the following: (1) the study was based on experiments with neonatal rodents; (2) hemorrhagic stroke models, models with global cerebral ischemia or hypoxic ischemia; and (3) the study was based on experiments with animals other than rodents. To diminish systematical bias, human stroke studies with less than 3 patients were excluded.

### Data Extraction

Two reviewers (CB and ASP) independently screened titles/abstracts of studies retrieved using the search strategy and extracted data from eligible studies into a standardized form. Any discrepancies were resolved by consensus or by consultation with a third reviewer (JM). Extracted data included animal stroke model, assessed region, time of cell count, analyzed cell type (lymphocytes, macrophages, or neutrophils), assessment method, immunostaining, cell quantification, infarct size, and the number of animals in the trial. When treatment was compared to control groups, only data of control groups were used. In histological analysis in rodent stroke models, the cell numbers were calculated as cells per mm^3^ and differences in the coronal section thickness were considered. Macrophages were identified by description of cell type within the different studies and cell staining. Studies using typical microglial marker, e.g., Iba1, were excluded. However, macrophages share several antigens with microglia cells, and thus, a reliable distinction between the different cell types is not always possible. Regarding fluorescence-activated cell sorting (FACS), only analyses of the ipsilateral brain hemisphere providing cell counts in cells per hemisphere were included. If CD4+ and CD8+ lymphocytes were mentioned separately, cell counts were added up to the total count of lymphocytes. The same applies for addition of M1 and M2 macrophages. For purpose of comparison, infarct volumes were only extracted in histological studies. If infarct volumes were reported as percentage of contralateral hemisphere, data were calculated in mm^3^ considering standard brain volume of mice [19–22] and rats [23–25]. Standard brain volumes were calculated from different mice and rat strains. When data were presented only graphically, values were read off the graphics using Adobe Acrobat X Pro (Adobe Systems; San Jose, CA).

In human stroke studies, obtained data included the number of patients in the trial, brain tissue source, days after stroke onset, analyzed cell type, analyzed region (e.g., ischemic core or penumbra), quantification/description of immune cell infiltration, and clinical information.

### Statistical Methods

We performed meta-analyses to illustrate the spatiotemporal distribution of immune cell infiltration in experimental stroke. We extracted mean values and their standard errors (SEs) for immune cell infiltration at a certain point of time after stroke induction. If not provided directly, the SE was computed by dividing the standard deviation by the square root of the number of animals per group. A meta-analysis could only be performed for rodent studies. Human studies often provided semiquantitative values for cell counts and therefore did not allow calculations with means of meta-analysis. Random-effects meta-analysis (DerSimonian-Laird method) was conducted using the metafor package in R (version 3.5.0). In order to quantify the heterogeneity of the collected data, *I*^2^ has been calculated for each item under investigation. Meta-regression analysis was performed after logarithmic transformation of the mean number of immune cell population and mean infarct volume values in mice and rats using “metareg” function in the “meta” package in R. In all analyses, a value of *p* < 0.05 was considered to represent a significant difference.

## Results

### Included Studies and General Study Characteristics

Our initial search of the literature and reference lists of included studies yielded a total of 17,463 studies (Fig. S1). Of these, 10,387 duplicates were removed, and 7076 records were screened for eligibility through title and abstract review. We excluded 6851 records that were not relevant to the research objectives; these articles, for example, reported on heat stroke or global/hypoxic cerebral ischemia. Following a thorough review of the full-text articles and after quality assessment, our search yielded 225 studies, of which 205 were eligible for inclusion: 188 studies in mice and rats and 20 human studies (with an overlap of 3 studies that reported outcomes for both animals and humans).

Outcomes in experimental studies were assessed in 120 studies for infiltration of neutrophils, 92 studies for infiltration of macrophages, 49 studies for infiltration of lymphocytes, and 126 studies for infarct size (Table S1). In human studies, outcome was assessed in 11 studies for infiltration of neutrophils, 13 studies for infiltration of macrophages (in 2 studies microglia/macrophages), and 7 studies for infiltration of lymphocytes.

### Immune Cell Infiltration in Human Stroke

Baseline characteristics of included human stroke studies are shown in Table 1. Some of the human studies provided clinical data on age (17 studies), sex (15 studies), vascular territory (11 studies), stroke etiology (4 studies), and cause of death (4 studies). The mean age of patients with stroke was 73.5 years (standard deviation, SD 7.8). The proportion of women was 55%. The middle cerebral artery territory was the most commonly affected vascular territory. The data of immune cell infiltration in human stroke were heterogeneous **(**Table 2**)**. Three studies provided a mere descriptive analysis of immune cell infiltration. In eight studies, a semiquantitative scoring system was applied to describe the histological findings and to evaluate the intensity or extent of the inflammatory response. Nine studies reported numerical data of immune cells within the infarcted tissue. Time period from onset of stroke to death varied between a few hours and several months. However, most studies did not report the explicit interval between stroke and death.

**Table 1:** Baseline characteristics of included human stroke studies

Study	*n*	Brain tissue source	Onset of stroke to death mean (range)	Assessed region	Evaluated cells (assessment method)	Age (years) mean ± SD (range)	% female	Vascular territory	Stroke etiology	Cause of death
Sörnäs [26]	5	Autopsy	4,4 days (2–6 days)	Infarcted area	Neutrophils (hematoxylin-eosin staining)	77 (69–85)	NS	NS	NS	NS
Barcikowska-Litwin et al. [27]	17	Autopsy	83 days (5 days–> 3 years)	Infarcted area	Macrophages NS	78 (50–87)	60	NS	NS	Pulmonary embolism (*n* = 8), myocardial infarction (*n* = 2), pneumonia (*n* = 5)
Esiri and Morris [28]	3	Autopsy	Recent or old	Lesion core and	Macrophages (Mac387 IHC, KP1 IHC)	NS (NS)	NS	NS	NS	NS
Chuaqui and Tapia [29]	30	Autopsy	8,7 days (16 h–27 days)	Perilesional area	Macrophages, neutrophils NS	65 (44–83)	67	ACM (*n* = 16), ACA (*n* = 4), PCA (*n* = 6), cerebellum (*n* = 10), brainstem (*n* = 8)	Embolic (*n* = 21), thrombotic (*n* = 9), secondary vasospasm (*n* = 1)	NS
Krupinski et al. [30]	10	Autopsy	NS (3 days–17 days)	Lesion core and	Macrophages (CD68 IHC)	NS (51–81)	NS	Left MCA	NS	NS
Lindsberg et al. [31]	11	Autopsy	6 days (15 h–18 days)	Perilesional area	Neutrophils (CD15 IHC)	67 (46–79)	45	ACI (*n* = 5), MCA (*n* = 2), BA (*n* = 3), OLA (*n* = 1)	NS	Stroke-related events (i.e., severe brain edema; *n* = 5), pulmonary embolism (*n* = 4), cardiac failure (*n* = 2)
Postler et al. [32]	18	Autopsy	NS (<24 h–months)	Perilesional area	Macrophages (CD68 IHC)	66 ± 15,1 (52–86)	67	MCA (*n* = 10), PCA (*n* = 6), BA (*n* = 2)	NS	NS
Schwab et al. [33]	20	Autopsy	NS (1 day–months)	Infarcted area	Macrophages, neutrophils (NS)	79 (52–87)	60	MCA (*n* = 10), PCA (*n* = 7), BA (n = 3)	NS	NS
Beschorner et al. [34]	18	Autopsy	NS (1 day–months)	Lesion core and perilesional area	Microglia/macrophages (CD14 IHC)	70 (52–87)	61	MCA (*n* = 7), PCA (*n* = 7), BA (*n* = 4)	NS	NS
Mena et al. [35]	137	Autopsy or surgical material	NS (1 day–53 years)	Infarcted area	Macrophages, neutrophils (NS)	67 (7–93)	24	Cerebral (*n* = 129), cerebellar (n = 3), brainstem (*n* = 3)	NS	NS
Mărgăritescu et al. [36]	22	Autopsy	NS (1 day–53 years)	Infarcted area	Macrophages, neutrophils (CD68 IHC)	62 (27–91)	32	Left MCA (*n* = 8), right MCA (*n* = 6), left ACI (*n* = 1), right ACI (*n* = 1), left ACA (*n* = 1)	NS	NS
Yilmaz et al. [37]	29	Autopsy	NS (< 24 h–> 4 h)	Infarcted area	Lymphocytes (CD3 IHC)	NS (NS)	NS	NS	NS	NS
Arsene et al. [38]	21	Autopsy	35,4 h (6 h–11,7 days)	Lesion core and	Lymphocytes, macrophages, neutrophils (CD20/L26 IHC, UCHL-1 ICH, CD68 IHC, CD15 IHC)	74 ± 14,4 (18–86)	48	MCA (*n* = 11), ACA (*n* = 3), ACI (*n* = 1), mixed localization (MCA and PCA; *n* = 1), BA (*n* = 5)	Large vessel thrombosis or cardioembolic mechanism	NS
Holfelder et al. [39]	30	Autopsy	NS (6 h–2,6 years)	Perilesional area	Microglia/macrophages (CD163 IHC)	68 (32–86)	63	NS	NS	NS
Enzmann et al. [40]	25	Autopsy or surgical material	NS (< 48 h -chronic stage stroke)	Infarcted area	Macrophages, neutrophils (CD68 IHC, CD15 + MPO + chloroacetate esterase IHC)	65 (45–86)	52	NS	NS	Cerebral infarction (*n* = 7), myocardial infarction (*n* = 3), aortic bleeding (*n* = 1), pulmonary bleeding (*n* = 1), heart failure (*n* = 4), circulatory arrest (*n* = 2), pneumonia (*n* = 2), other (*n* = 5)
Perez-de-Puig et al. [41]	3	Autopsy	NS (1 day–5 days)	Lesion core and perilesional area	Neutrophils (neutrophil elastase staining)	85 (79–89)	67	Left MCA (*n* = 1), BA (*n* = 2)	Cardioembolic (*n* = 2), large vessel disease (*n* = 1)	NS
Nguyen et al. [42]	7	Autopsy	Stage of liquefactive necrosis	Infarcted area	Lymphocytes (CD4 IHC, CD8 IHC, CD20 IHC)	87 (77–106)	NS	NS	NS	NS
Feng et al. [43]	5	Autopsy	NS (7 days–14 days)	Infarcted area	Lymphocytes (CD4 ICH, CD8 ICH)	76,2	60	NS	NS	NS
Li et al. [44]	6	Autopsy	NS (3 days–7 days)	Perilesional area	Lymphocytes (CD8 IHC)	82 ± 9(SEM)	33	MCA (*n* = 6)	NS	NS
Zrzavy et al. [45]	16	Autopsy	NS (1 day–240 days)	Lesion core and perilesional area	Lymphocytes, macrophages, neutrophils (CD3 IHC, CD68 IHC, p22phox IHC)	81,06 ± 10,1 (66–97)	56	NS	Small-vessel occlusion (*n* = 3), large-artery atherosclerosis (*n* = 3), cardio embolism (*n* = 3), other (*n* = 3), undetermined (*n* = 4	Cardiac arrest (*n* = 2), respiratory failure (*n* = 2), cardiopulmonary failure (*n* = 2), heart failure (*n* = 2), pneumonia (*n* = 2), other (*n* = 1), NS (*n* = 5)

ACA, anterior cerebral artery; ACI, internal carotid artery; MCA, middle cerebral artery; OLA, occipital lobe artery; PCA, posterior cerebral artery; BA, basilar artery; IHC, immunohistochemistry, SD, standard deviation; NS, not specified

**Table 2:** Spatiotemporal distribution of immune cells in human stroke studies

Study	Analyzed region	Days after stroke onset	1	2	3	4	5	6	7	7–14	14–28	> 28
		Measure	Cell quantification or description of immune cell infiltration (number of analyzed patients, *n*)									
			**Lymphocytes**									
Feng et al. [43]	Infarcted area	Mean ± SEM (cells/mm^2^)								26,16 ± 4^a^, n = 5		
Feng et al. [43]	Infarcted area	Mean ± SEM (cells/mm^2^)								57,46 ± 8,03^b^, n = 5		
Li et al. [44]	Peri-infarct region	Mean ± SEM (cells/mm^2^)					112,644 ± 36,18^a^, n = 6					
Zrzavy et al. [45]	Infarcted area	Median (cells/mm^2^)				3,73^d^, n = 9						
Nguyen et al. [42]	Infarcted area	Mean ± SEM (cells/mm^2^)										4,03 ± 3,04^a^, n = 7
Nguyen et al. [42]	Infarcted area	Mean ± SEM (cells/mm^2^)										1,71 ± 1,16^b^, n = 7
Nguyen et al. [42]	Infarcted area	Mean ± SEM (cells/mm^2^)										1,43 ± 2,21^c^, n = 7
Yilmaz et al. [37]	Infarcted area	Mean ± SEM (cells/mm^2^)	54,4 ± 10,48^d^,									30,36 ± 6,12^d^, n = 12
Arsene et al. [38]			No lymphocytes accumulated adjacent to the infarcted area or remote to it, n = 21									
Krupinski et al. [30]			Numerous single lymphocytes accumulate around blood vessels, n = 10									
			**Neutrophils**									
Zrzavy et al. [45]	Ischemic core	Median (cells/mm^2^)	30,65, n = 9									
Zrzavy et al. [45]	Peri-infarct region	Median (cells/mm^2^)	211,58, n = 9									
Perez-de-Puig et al. [41]	Ischemic core, perivascular	Mean ± SEM (cells per area in brain sections)	0,13 ± 0,13, n = 3									
Perez-de-Puig et al. [41]	Penumbra, perivascular	Mean ± SEM (cells per area in brain sections)	0, n = 3									
Perez-de-Puig et al. [41]	Ischemic core, extravascular	Mean ± SEM (cells per area in brain sections)	0,16 ± 0,12, n = 3									
Perez-de-Puig et al. [41]	Penumbra, extravascular	Mean ± SEM (positive cells per area in brain sections)	0,23 ± 0,12, n = 3									
Lindsberg et al. [31]	Ischemic core	Mean (cells/mm^2^)	5,32, n = 1	51,48, n = 3	2,15, n = 1	14,53, n = 1	0, n = 1	3,14, n = 1		2,41, n = 1		
Lindsberg et al. [31]	Peri-infarct region	Mean (cells/mm^2^)	11,61, n = 1	42,5, n = 3	106,98, n = 1	9,2, n = 1	29,79, n = 1	0,25, n = 1		4,84, n = 1		
Mărgăritescu et al. [36]	Infarcted area	Positive cases	1, n = 2		4, n = 20							
Mena et al. [35]	Infarcted area	Positive cases	6, n = 11		25, n = 126							
Chuaqui and Tapia [29]	Ischemic core and peri-infarct region	Median (range), degree of infiltration: none (−), mild (+), moderate (++), strong (+++))	+ (0 to ++), n = 3	+++ (++ bis +++), n = 2	++ (++ to +++), n = 3	+ (+ to ++), n = 3	++ (+ to ++), n = 2	+ (+), n = 1	+ (+), n = 2	0 (0 to +), n = 9	0 (0), n = 6	
Sörnäs [26]	Brain parenchyma	Median (range), degree of infiltration: none (−), mild (+), moderate (++), strong (+++))		+ (+), n = 1		+ (+), n = 1	++ (+ to ++), n = 2	++ (++), n = 1				
Enzmann et al. [40]	Infarcted area	Descriptive	Very few in early infarct stages and at stages of resorption, majority localized within the lumen of blood vessels or in the perivascular space, no neutrophils in the inner cortical layers or in the infarct center and border zones, neutrophils remained confined to vessel lumina, n = 25									
Arsene et al. [38]	Peri-infarct region	Descriptive	No polymorphonuclear cells accumulated adjacent to the infarcted area or remote to it, n = 21									
Schwab et al. [33]	Infarcted area	Descriptive	Rare, n = 1	Rare, n = 2 Moderate, n = 1	Moderate, n = 1	Moderate, n = 1						
Krupinski et al. [30]	Infarcted area	Descriptive	Numerous neutrophils accumulate around blood vessels, n = 10									
			**Macrophages (microglia)**									
Zrzavy et al. [45]	Ischemic core	Median (cells/mm^2^)	71,13, n = 9									
Zrzavy et al. [45]	Peri-infarct region	Median (cells/mm^2^)	11,63, n = 9									
Holfelder et al. [39]	Ischemic core	Median (cells/mm^2^)	1,65, n = 5	39,39, n = 10						968,21, n = 8		
Holfelder et al. [39]	Peri-infarct region	Median (cells/mm^2^)	6,56, n = 5	12,58, n = 10						227,55, n = 8		
Beschorner et al. [34]	Ischemic core	Mean ± SEM (cells/mm^2^)	43,2 ± 6,4, n = 18									
Beschorner et al. [34]	Peri-infarct region	Mean ± SEM (cells/mm^2^)	11,2 ± 1,6, n = 18									
Mărgăritescu et al. [36]	Infarcted area	Positive cases	0, n = 2	16, n = 20								
Mena et al. [35]	Infarcted area	Positive cases	0, n = 11	103, n = 126								
Postler et al. [32]	Peri-infarct region	Mean ± SD (cells/mm^2^)	54 ± 28,8, n = 10	92,4 ± 27,2, n = 8								
Chuaqui and Tapia [29]	Ischemic core and peri-infarct region	Median (range): slight (+), moderate (++), intense (+++)	0 (0), n = 3	0 (0), n = 2	0 (0), n = 3	0, n = 3	++, + bis ++, n = 2	+ (+), n = 1	+ (+), n = 2	++ (++ bis +++), n = 9	++ (++ bis +++), n = 6	
Esiri and Morris [28]	Ischemic core and peri-infarct region	Median (range): 1 = rare; 2 = few; 3 = many; 4 = numerous, recent or old (= several weeks) lesion	2 (2–4), n = 2								2 (1–3), n = 1	
Barcikowska-Litwin et al. [27]	Infarcted area	Median (range): small (+), moderate (++), severe number (+++)					+ (+), n = 1			++ (0 to +++), n = 6	++ (+ to +++), n = 6	+ (0 to +++), n = 4
Enzmann et al. [40]	Infarcted area	Descriptive	Mainly in the perivascular space or brain parenchyma, low amount of extravasated cells, n = 8									
Arsene et al. [38]	Ischemic core and peri-infarct region	Descriptive	Present in large number in the necrotic areas or immediately adjacent to these in long standing stroke cases higher amount in the penumbra than in the contralateral symmetric or remote, unaffected areas, n = 21									
Schwab et al. [33]	Infarcted area	Descriptive			Macrophages, n = 1	Macrophages, n = 3						Dense/moderate density, n = 4
Krupinski et al. [30]	Infarcted area	Descriptive	In the core of the infarct and the surrounding area, in the infarcted area numerous macrophages accumulate around blood vessels, n = 10									

SD, standard deviation; SEM, standard error of the mean. ^a^CD8+ lymphocytes; ^b^CD4+ lymphocytes; ^c^CD20+ lymphocytes ; ^d^CD3+ lymphocytes

Among the human studies reporting histological data, different monoclonal antibodies for immunohistochemical staining were used. The most common antibody for macrophages was anti-CD68. Lymphocytes and their subsets were determined with anti-CD3, anti-CD4, and anti-CD8 antibodies. The immunostaining of neutrophils varied widely (e.g., hematoxylin-eosin, CD15, or neutrophil elastase). Four studies did not report the type of staining. Descriptions and terminology of the analyzed region varied widely. The assessed region was defined as infarcted area, peri-infarct region, ischemic core, or brain parenchyma.

Overall, the number of lymphocytes, macrophages, and neutrophils was higher in the infarcted area compared to the peri-infarct region (Table 2). Macrophages accumulated in the early phase (day 1) after stroke onset within both the ischemic core and in smaller numbers within the peri-infarct region. Some studies report macrophage infiltration not until 3 days after cerebral ischemia with a peak 7 days after stroke onset. Microscopically, accumulation of macrophages was observed around blood vessels as well as in the perivascular space. In most studies, neutrophil infiltration peaked at days 2 and 3 after stroke onset. In contrast, Sörnäs et al. found a strong neutrophil infiltration 5–6 days after stroke and only a mild earlier infiltration [26]. Additionally, in 4 out of 11 studies analyzing the neutrophil infiltration, the vast majority of neutrophils were detected within the lumen of blood vessels and the perivascular or leptomeningeal space of the infarcted area [30, 40, 41, 45]. Enzmann and colleagues found neutrophils in rather low counts or even completely absent within the infarcted brain parenchyma in very acute lesions (< 48 h) [40]. Overall, the number of infiltrating lymphocytes was lower compared to macrophages and neutrophils (Table 2). However, due to the few and heterogenous data on lymphocytes, conclusions on their spatiotemporal distribution are limited.

### Immune Cell Infiltration in Rodent Stroke

**Fig. 1:**
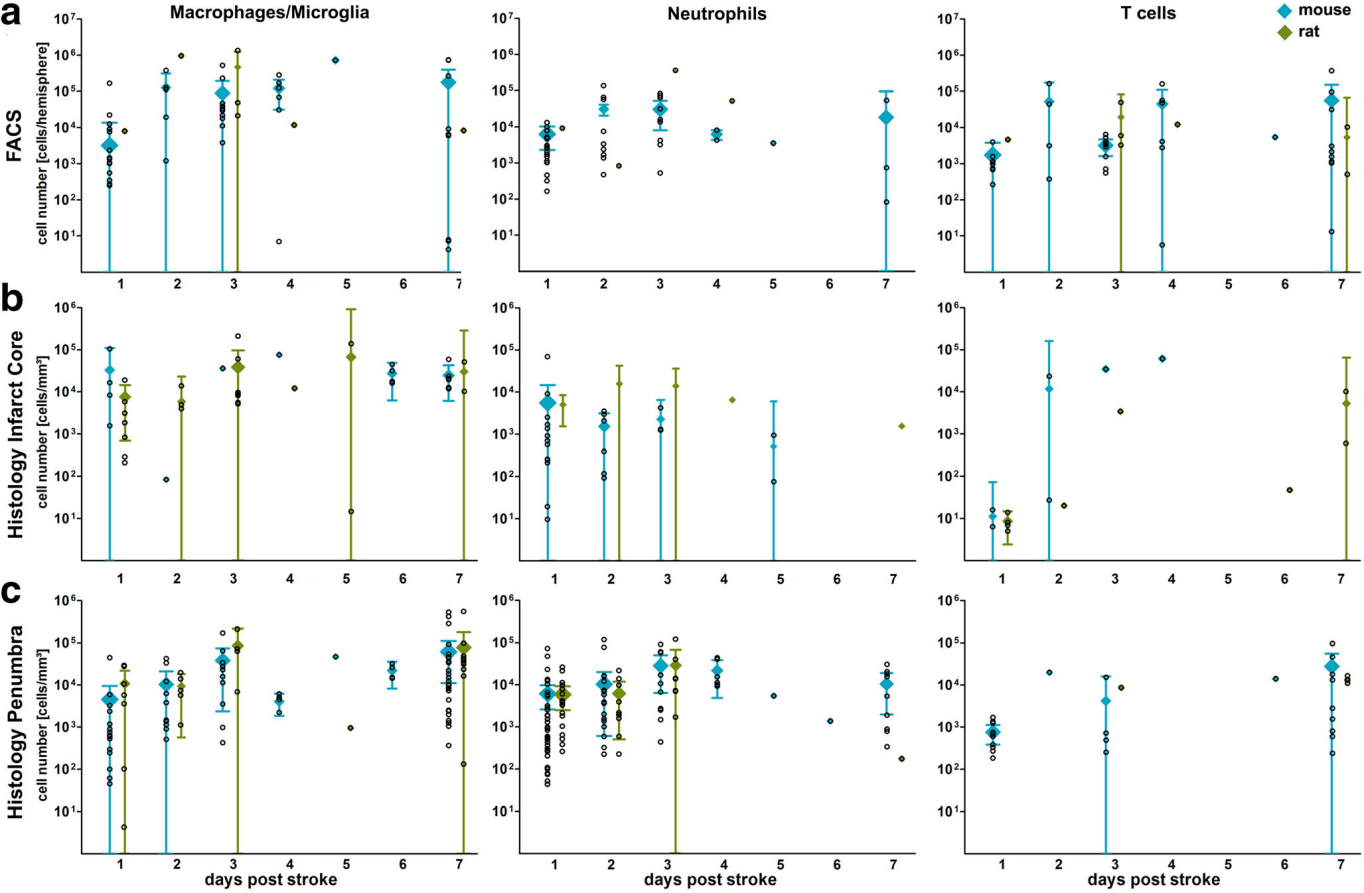
Temporal and quantitative characterization of immune cell infiltration in rodent stroke at days 1–7 after induction of ischemia. Flow cytometric analysis of the ipsilateral brain hemisphere (a) and histological analysis (b infarct core, c penumbra) showing the absolute numbers (bare circles) of infiltrating immune cells (macrophages, neutrophils, and T cells) in the ischemic hemisphere in rats (blue) and mice (green). Meta-analyzed data are shown as mean with higher and lower 95% confidence interval. In case of negative confidence intervals, lower confidence limits are not shown. For clarity, data are shown as data of 10 (logarithmic scale)

**Fig. 2:**
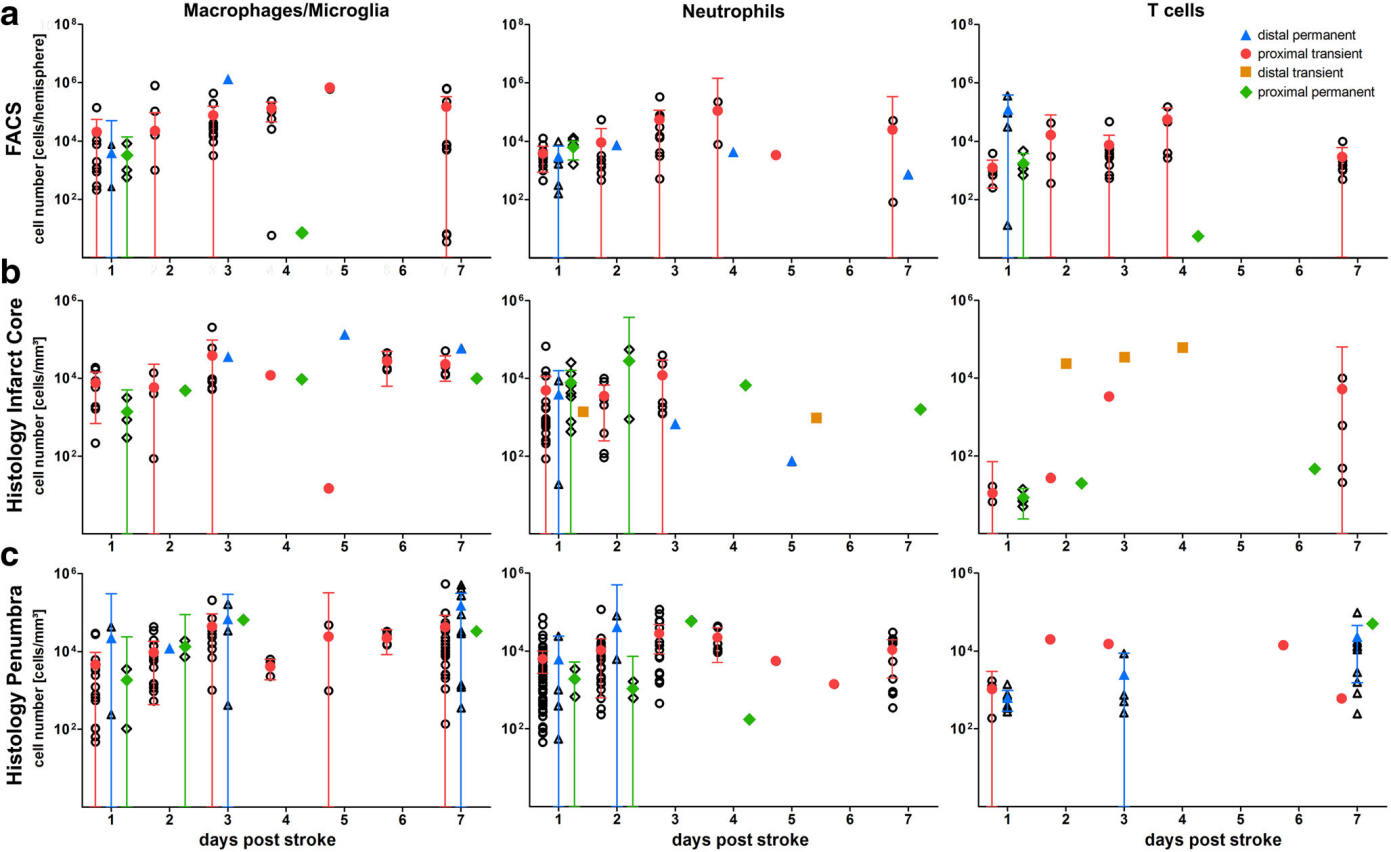
Immune cell infiltration in relation to animal stroke model at days 1–7 after induction of ischemia. Flow cytometric analysis of the ipsilateral brain hemisphere (a) and histological analysis (b infarct core, c penumbra) showing the absolute numbers of infiltrating immune cells (macrophages, neutrophils, and T cells) in the ischemic hemisphere for each type of ischemia: distal permanent (bare triangle), proximal transient (bare circle), distal transient (bare square), and proximal permanent (bare rhombus). Meta-analyzed data are shown as mean with higher and lower 95% confidence interval (red). In case of negative confidence intervals, lower confidence limits are not shown

Neutrophils started to appear within 24 h after ischemia and peaked at day 2 in FACS and at day 3 in histological analyses **(**Fig. 1a**)**. A noticeable decline of immigrated neutrophils occurred from days 4 to 7 **(**Fig. 1a**)**. Neutrophil counts within the infarct core seem to be higher in rats compared to mice, whereas there are slightly higher cell counts in mice within the penumbra **(**Fig. 1b, c**)**. The temporal profile of macrophage infiltration revealed an early influx with a peak at day 2 and a second peak at day 4/5(Fig. 1). Cell counts did not decrease until day 7 after stroke induction. In the penumbra, macrophage cell counts seem to be slightly elevated in rats in relation to mice **(**Fig. 1c**)**. Comparing different experimental stroke models showed that on days 1 and 7 poststroke, the cell count of infiltrated macrophages was lower in proximal permanent in contrast to proximal transient stroke models **(**Fig. 2a–c**)**. The same applies for the number of neutrophils within the penumbra on days 1 and 2 poststroke **(**Fig. 2c**)**. Interestingly, on days 2 and 3, this difference disappears, and cell counts are nearly identical with a slight tendency towards higher cell counts in proximal permanent stroke models **(**Fig. 2c**)**. In distal permanent stroke models, cell counts of macrophages and neutrophils seem to reach the highest peak numbers in the peri-infarct region compared to other stroke models **(**Fig. 2c**)**. This finding holds not true for cell counts in FACS analysis and infarct core **(**Fig. 2a, b**)**. However, comparison of different stroke models and the comparison of animals and patients may be limited by the preponderance of data from proximal transient stroke models.

T cells appeared in small amounts within 24 h and slightly increased until day 4 **(**Fig. 1a–c**)**. The peak number of T cells on the first 2–4 days poststroke was nearly identical to the amount at day 7, indicating that T cells reach a plateau in ischemic tissue **(**Fig. 1a, c**)**. In contrast, histological analysis of the infarct core showed a peak of infiltration on day 4 **(**Fig. 1b**)**. Analyses of distinct experimental stroke models did not show any significant differences regarding the amount and distribution of infiltrating T cells (Fig. 2).

We found that macrophages were the most numerous cell type infiltrating the brain throughout the first 72 h and particularly at day 7 after induction of cerebral ischemia (Fig. 1). However, there were higher numbers of neutrophils on day 2 detected by FACS analysis **(**Fig. 1a**)**. Our results indicate that the infiltration of the ischemic hemisphere by macrophages and neutrophils precedes the lymphocytic influx. Interestingly, immunohistochemical analysis revealed comparable peak cell counts of macrophages and T cells within the infarct core compared to the peri-infarct region (Fig. 1b, c).

**Fig. 3:**
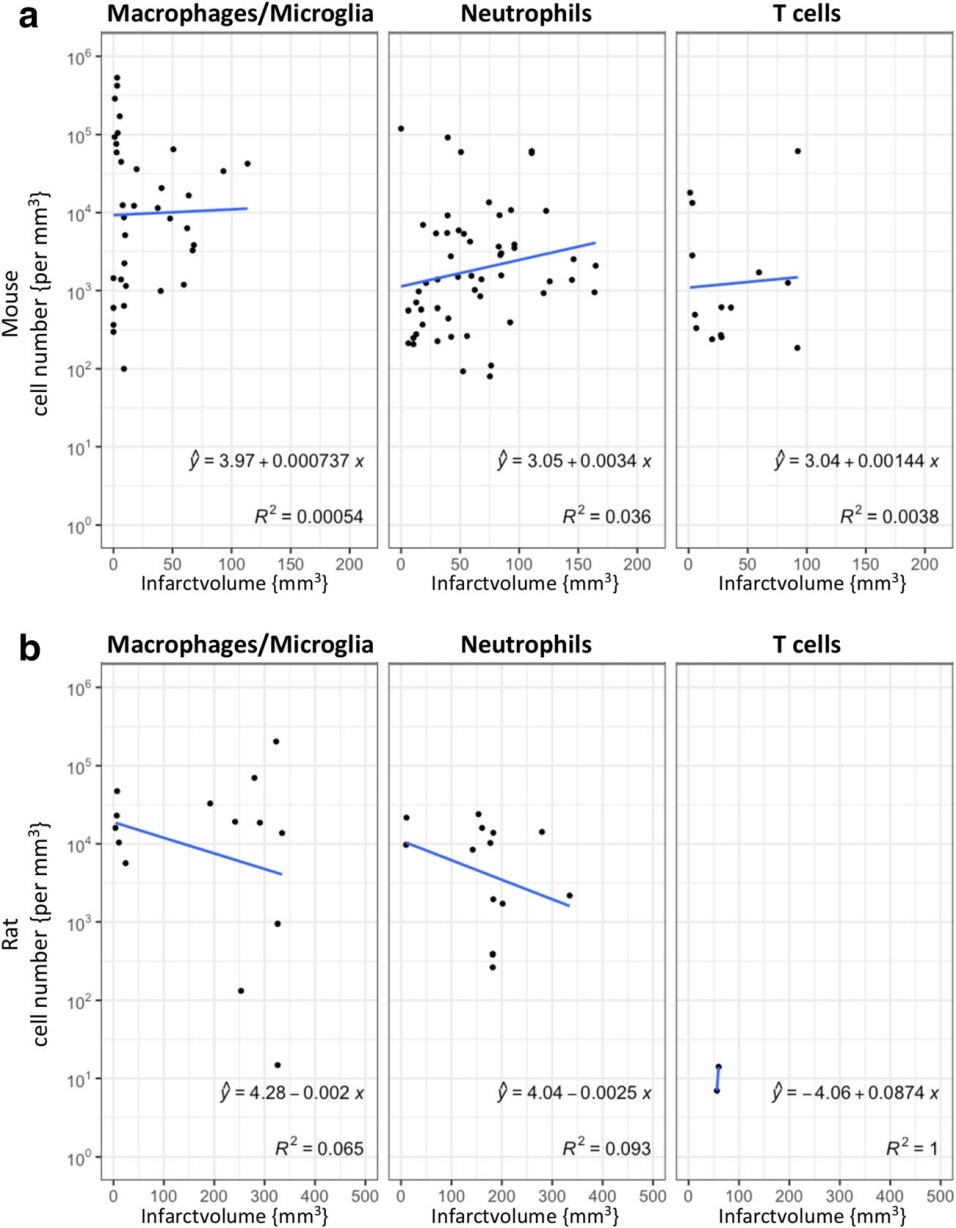
Meta-regression analysis of the association between immune cell density of macrophages/microglia, neutrophils, or T cells and infarct volume 1–42 days after induction of ischemia. Due to the significant difference in infarct size, values for mice (a) and rats (b) are calculated separately. Values represent numbers of immune cells/mm^3^ in the infarct core and infarct volume in mm^3^

We next performed a correlation analysis of the density (cells/mm^3^) of infiltrated immune cells with the infarct volume **(**Fig. 3**)**. Meta-regression analysis did not indicate any significant association between immune cell density and infarct volume.

### Summary and Comparison of Immune Cell Infiltration in Human and Rodent Stroke

**Fig. 4:**
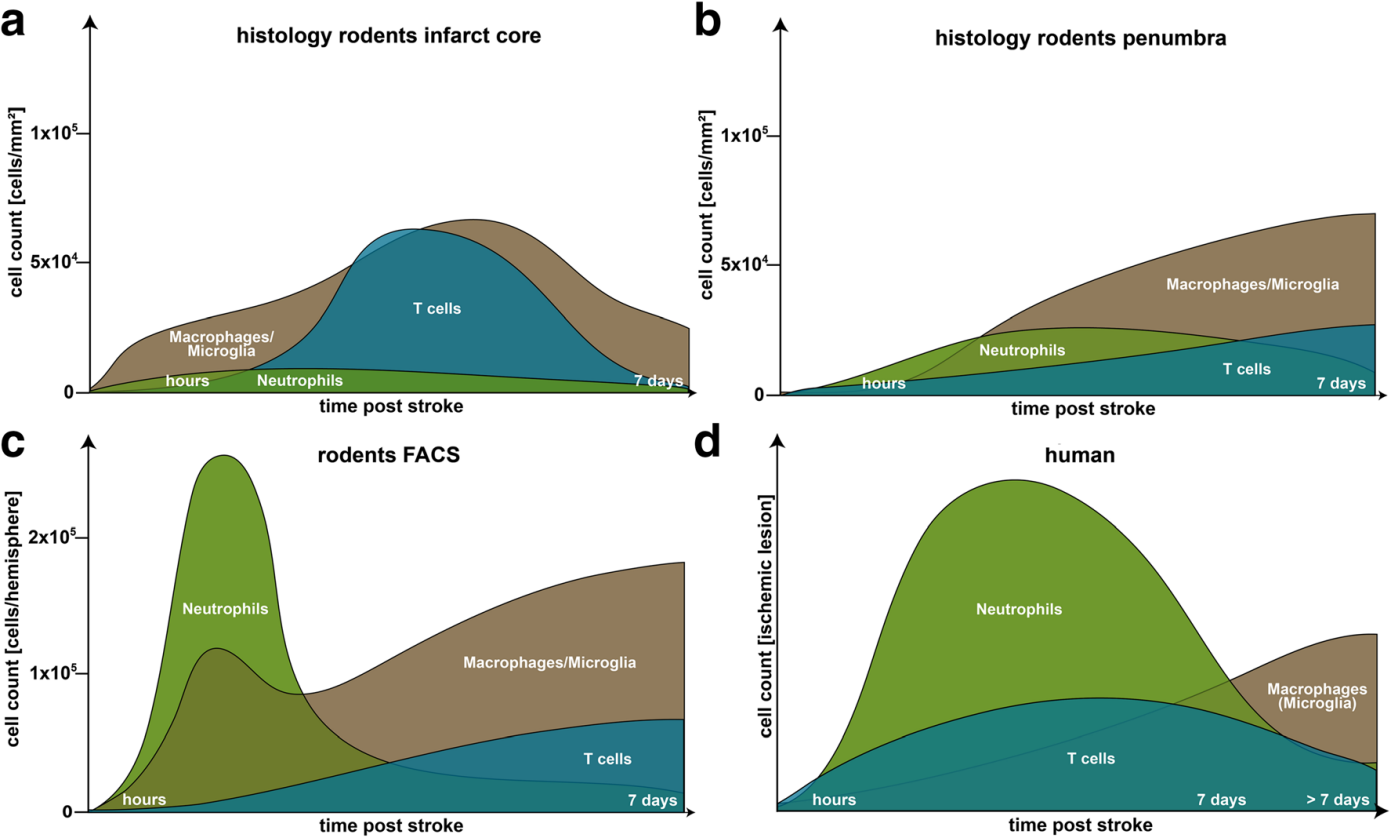
Schematics of temporal profile of immune cell infiltration in rodent (a, b, c**)** and human (b) stroke. Curves are created from data obtained from this study. In rodent stroke, numbers of immune cells are graphically calculated from the original data of histological (cells/mm^3^; a, b) and of FACS analysis (cells per ischemic hemisphere; c)

Based on the analyses of this study, we summarized our findings on the temporal infiltration of immune cells in humans and rodents as shown in Fig. 4. Overall, the temporal dynamics of post-ischemic neuroinflammation is comparable in humans and rodents. However, there are certain findings that need to be elucidated. First, in experimental stroke, both cell count and relative distribution of immune cells are determined by the modality of analysis (histology vs. FACS) used. In FACS analysis, neutrophils were the most abundant immune cells within the first days after stroke induction **(**Fig. 4c**)**. Furthermore, FACS analysis revealed a dual-wave-like infiltration of macrophages different to the slight increase and rather late peak in histological analysis. Second, comparison of infarct core and penumbra in histological animal studies demonstrates significant differences regarding the proportion of immune cell subsets after cerebral ischemia **(**Fig. 4a, b**)**. For instance, the increase of T cells is more pronounced in the infarct core than in the penumbra. Besides differences within experimental stroke studies, our study identifies a temporal distribution pattern in human stroke slightly distinct from the immune cell response in rodent stroke studies **(**Fig. 4d**)**. Of note, macrophages are rather slightly increasing in human stroke lesions suggesting a less pronounced role in the early phase poststroke. Nevertheless, it must be taken into account that the early and high peak of macrophage infiltration in human stroke might be explained by histological staining that potentially includes microglia.

## Discussion

In this systematic review and meta-analysis, we summarize data on post-ischemic neuroinflammation from 188 rodent and 20 human studies. Our analyses yield the following main findings: (1) temporal dynamics of immune cell infiltration is comparable after human and rodent stroke; (2) in rodent stroke, post-ischemic immune cell infiltration by macrophages and neutrophils preceded the lymphocytic influx and macrophages and neutrophils were the most numerous immune cell subtypes within 72 h after the onset of ischemia; (3) immune cell infiltration was highly heterogenous across human as well as rodent studies; and (4) counts of macrophages and in part of neutrophils are higher in proximal transient compared to proximal permanent stroke models on days 1 and 7 after ischemia.

Although the post-ischemic neuroinflammation of major immune cell subsets is comparable between rodents and humans, the translation of experimental anti-inflammatory stroke therapies into an effective treatment for patients has so far not been successful. Our study provides possible explanations for this translational failure. We identified heterogenous findings among the immune cell infiltration across rodent stroke studies. For instance, after proximal transient (60 min) ischemia in mice macrophage counts in FACS, analyses were threefold higher at 24 h in some studies compared to others. Counts of neutrophils were 2.5 times higher in proximal permanent compared to proximal transient stroke models in mice 24 h after stroke induction. Besides discrepancies regarding the absolute cell counts at certain time points after stroke induction, we also identified differences in the peak of immune cell infiltration. For instance, in some studies, neutrophils already peak 48 h after stroke induction, whereas others describe the peak of neutrophilic influx at 72 h after infarction. In addition, the role and significance of certain types of immune cells in ischemic stroke is controversial. For example, while B cells were recently reported to have a potential neuroprotective function in murine experimental stroke [46], separate studies could not confirm this observation suggesting that B cells play a lesser role in ischemic stroke [47, 48]. The same applies for studies on the effects of unselective macrophage depletion after stroke. A recent study showed that both selective and unselective monocyte/macrophage depletion and macrophage transfer did not influence tissue damage in the acute phase after experimental stroke, whereas different studies revealed beneficial as well as detrimental effects [49–51]. The discrepancies in experimental poststroke immune response could be a potential reason why in certain animal models immunomodulatory agents are beneficial and in others not. Apart from the differences in experimental setup and post-ischemic neuroinflammation in murine stroke models, heterogenous results due to different types of stroke, i.e., with or without recanalization, proximal or distal vessel occlusion, gray and/or white matter affected, can also be found in human stroke. Hence, these different conditions in human stroke, which cannot be validly represented by a certain experimental stroke model, lead to distinct results in poststroke immune cell response. Considering heterogeneity across human studies, detailed stroke characteristics are either missing or highly variable. For instance, the localization of the ischemic lesion (white vs. gray matter; forebrain vs. cerebellar vs. brain stem infarction), that is highly relevant for the clinical syndrome and functional outcome, is either not considered in the analysis of neuroinflammatory response or even merely reported. Another aspect is that occlusion pattern differs between animal models and patients with stroke. In particular, transient proximal occlusions are mainly performed in animal models, whereas patients more often have permanent proximal but also permanent distal occlusions. Furthermore, it is worth noting that in experimental stroke studies, the main focus is frequently on infarct size, although this reflects reality in human conditions only to a limited extent. In human stroke, the localization of the ischemic lesion within particular connections may be more relevant for the clinical symptoms and functional outcome. Furthermore, animals predominantly used in experimental stroke studies are young and healthy, whereas the typical stroke patient is aged and comorbid. Modeling stroke-associated risk factors, such as hypertension, diabetes, and hyperlipidemia, is important to model the immune system in human stroke given they profoundly affect the immune system and functional recovery. Experimental studies with aged animals demonstrated that neurological impairment increases, whereas the regenerative capacity is lowered compared to younger animals [52]. Moreover, preclinical studies on animals of both sexes in order to identify sex-based differences are lacking. Sex is known to display an important factor significantly affecting stroke incidence and outcome [53]. A potential solution to this dilemma could be the inclusion of selected, homogenous stroke patients with similarities to animal stroke studies and vice versa. In this context, noninvasive inflammation imaging in order to identify homogenous stroke patients with a proven local inflammatory response might support patient selection for clinical studies [54].

Our study has strengths and limitations. First, data on poststroke neuroinflammation in patients are mainly derived from small studies in which only a few time points after stroke were evaluated. In addition, some human studies provided only semiquantitative analyses of immune cells. Therefore, human studies could not be summarized by means of meta-analysis. A further limitation of our study is the focus on the acute phase after stroke, while it was demonstrated that the inflammatory response also affects long-term recovery. However, only a few studies on inflammatory changes later than 7 days after stroke were published which do not allow using meta-analysis techniques. Moreover, due to the small sample size and heterogeneity, statistical significance could not be determined for comparison between groups and hence analysis was based on visual interpretation of the graph. Nevertheless, a strength of our study is the large number of included animal studies allowing a more comprehensive analysis of temporal and spatial dynamics of poststroke neuroinflammation compared single studies.

## Conclusion

In summary, this systematic review and meta-analysis represents the first systematic analysis and comparison of human and rodent studies on post-ischemic neuroinflammation. Basically, the inflammatory response in rodent stroke models is comparable to that in patients with stroke. However, the heterogeneity of the post-ischemic immune response depending on the duration and location of the vessel occlusion and the mode of ischemia induction might contribute to the translational failure in stroke research. Therefore, stroke patients selected for future studies should be more homogenous and better comparable to animal models in corresponding experimental studies.

## Supplementary Information

ESM 1 (DOCX 21 kb)
