# Supplementary material for: Immune Cell Infiltration into the Brain After Ischemic Stroke in Humans Compared to Mice and Rats: a Systematic Review and Meta-Analysis
Source: Transl Stroke Res. 2021 Jan 26;12(6):976–90. doi: 10.1007/s12975-021-00887-4 (PMC8557159; doi:10.1007/s12975-021-00887-4)
Supplement: Supplementary file 1 — (DOCX 21 kb) [file 12975_2021_887_MOESM1_ESM.docx]

| **Table S1: Animal Studies of immune cell infiltration in Focal Cerebral Ischemia** | | | | | | | |  |  |  |  |  |  |  |
| --- | --- | --- | --- | --- | --- | --- | --- | --- | --- | --- | --- | --- | --- | --- |
| **Evaluated cells** | **All studys, n** | **Species** | | **IHC, n** | **FACS, n** | **Stroke model, n** | | | |  | **Days after stroke onset, n** | | | |
|  |  | Mice | Rat |  |  | proximal transient | proximal permanent | distal transient | distal permanent |  | d1 | d2-3 | d4-5 | d6-7 |
|  |  |  |  |  |  |  |  |  |  |  |  |  |  |  |
| Neutrophils | 120 | 82 | 38 | 90 | 30 | 99 | 9 | 1 | 11 |  | 66 | 73 | 14 | 12 |
| Macrophages/ Microglia | 92 | 56 | 36 | 60 | 32 | 73 | 8 | 0 | 11 |  | 37 | 56 | 19 | 32 |
| T-Lymphocytes | 49 | 38 | 11 | 16 | 33 | 34 | 7 | 1 | 7 |  | 20 | 26 | 7 | 19 |
| FACS, fluorescence-activated cell sorting; IHC, immunohistochemistry. | | | | | | |  |  |  |  |  |  |  |  |
